# Supplementary material for: Metabolic requirements of NK cells during the acute response against retroviral infection
Source: Nat Commun. 2021 Sep 10;12:5376. doi: 10.1038/s41467-021-25715-z (PMC8433386; doi:10.1038/s41467-021-25715-z)
Supplement: Supplementary file 1 — Supplementary Information [file 41467_2021_25715_MOESM1_ESM.pdf]

Supplementary Figure S1

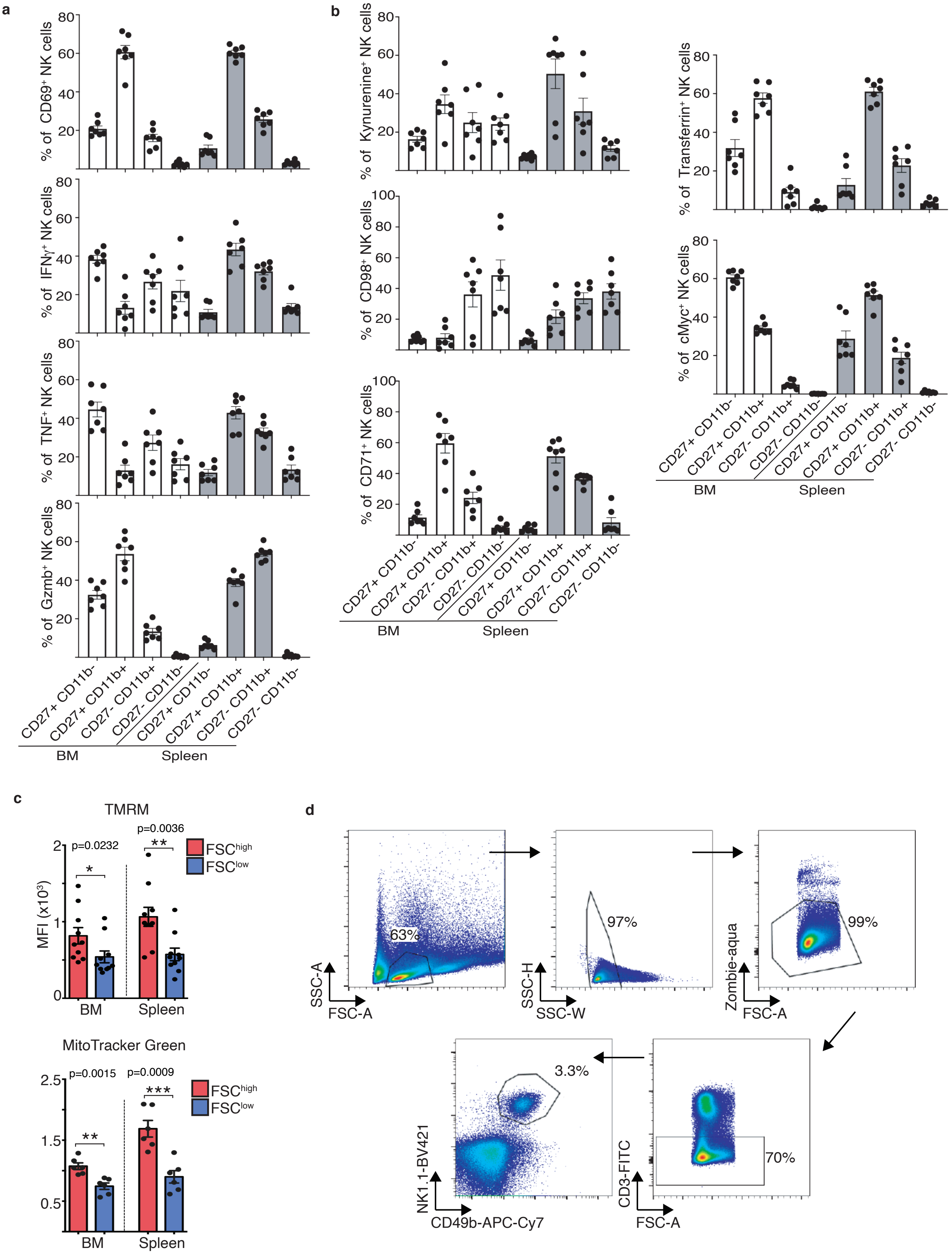

Supplementary Figure S1: Subset distribution of NK cells and mitochondrial capacity of FSC^{high} and FSC^{low} NK cells upon FV infection

C57BL/6 mice were FV-infected for 7 days. Single cell suspensions from bone marrow (BM) and spleens were prepared and NK cells were stained for the indicated effector functions (a) and metabolic markers (b). These population were further analysed for CD27 and CD11b subset distributions. Single cell suspensions were stained for the mitochondrial markers Tetramethylrhodamine, methyl ester (TMRM) and MitoTrackerGreen (MTG) (c). NK cells were gated on lymphocytes, singlets, viable cells, non-T cells (CD3^{-}) and NK1.1^{+} CD49b^{+} NK cells (d). We used at least six mice per group from two independent experiments. Statistically significant differences between the FSC^{high} and FSC^{low} group in the bone marrow or spleen were analysed with a two-tailed Mann-Whitney test and are displayed as *$p < 0.05$ and **$p < 0.01$. All data are presented as mean values +/- SEM. Source data are provided as a Source Data file.

Supplementary Figure S2

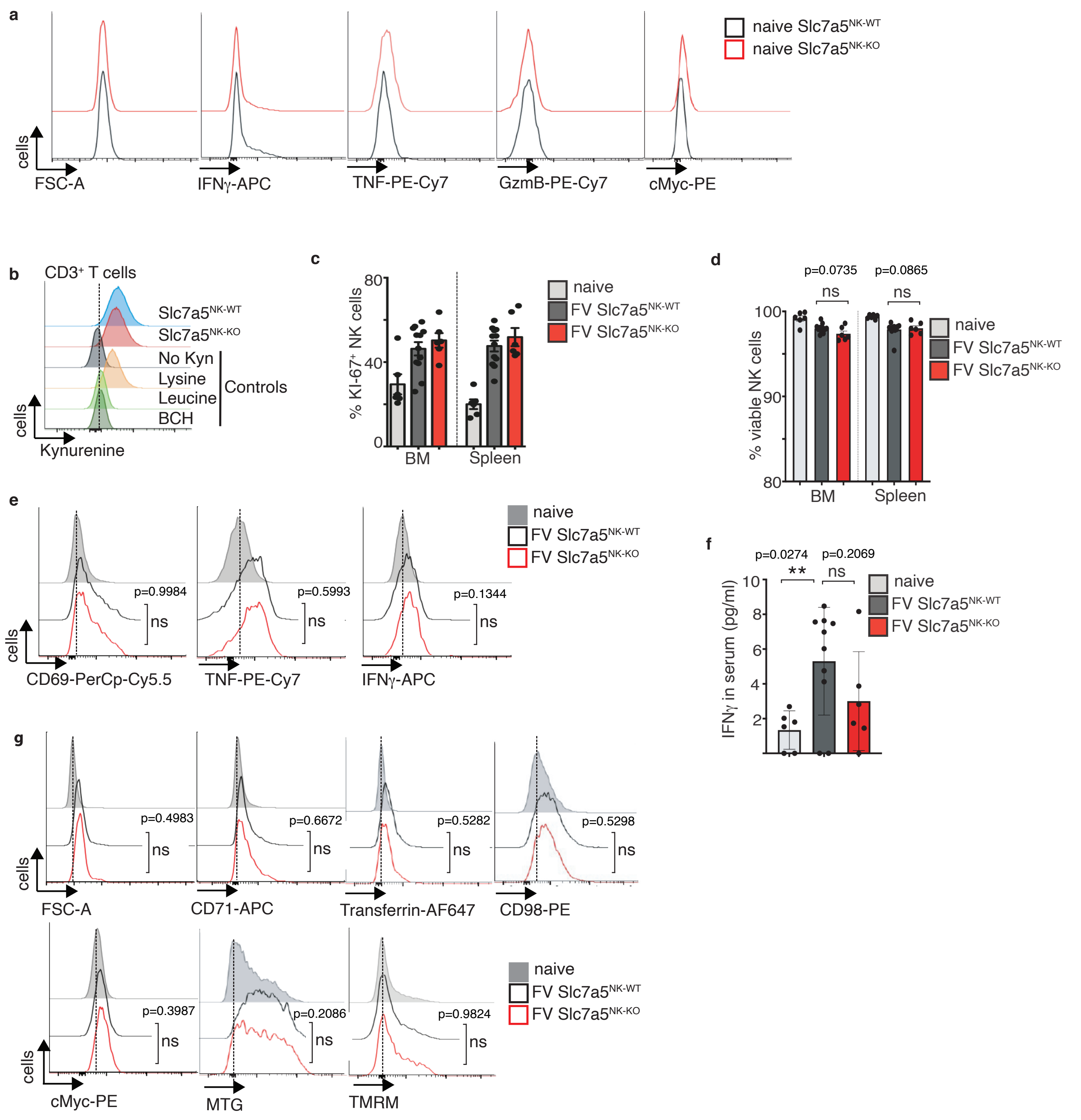

Supplementary Figure S2: Specificity and NK cell proliferation of Slc7a5$^{NK\text{-}WT}$ and Slc7a5$^{NK\text{-}KO}$ mice

At steady state, NK cells from Slc7a5$^{NK\text{-}WT}$ (black line) and Slc7a5$^{NK\text{-}KO}$ (red line) mice were analysed for cell size (FSC-A), IFNγ, TNFα, granzyme B and cMyc expression. Representative histograms are shown in a. Experiments were repeated independently twice with similar results. Splenocytes of Slc7a5$^{NK\text{-}WT}$ and Slc7a5$^{NK\text{-}KO}$ mice were gated for T cells (lymphocytes, singlets, viable cells CD3^{+} cells) and analysed for the expression of kynurenine in controls (no kynurenine, lysine, leucine, BCH) in FV-infected Slc7a5$^{NK\text{-}WT}$ (blue line) and Slc7a5$^{NK\text{-}KO}$ (red line) mice (b). Proliferation was detected by measuring KI-67^{+} NK cells in the spleen and bone morrow (BM, c). d) The viability of NK cells was analysed with a viability detection dye (Zombie, BioLegend). In e, representative histograms for CD69, TNFα and IFNγ of splenic NK cells from naïve mice (grey) and FV-infected Slc7a5$^{NK\text{-}WT}$ (black) or FV-infected Slc7a5$^{NK\text{-}KO}$ mice (red) are shown. Experiments were repeated independently twice with similar results. In f, the IFNγ concentration in mouse serum was analysed by Legendplex assay in naïve and mice infected for 3 days with FV. At least six mice per group from two independent experiments were used. Statistically significant differences between the groups were analysed with an Ordinary one-way ANOVA (e-g). Metabolic measurements such as cell size (FSC-A), transferrin receptor expression (CD71), Transferrin uptake, CD98, cMyc expression as well as MitoTrackerGreen and TMRM are displayed as representative histograms in g. Experiments were repeated independently twice with similar results. *$p < 0.05$ and ***$p < 0.001$. Data are presented as mean values +/- SEM (c, f, f). Source data are provided as a Source Data file. ns = not significant

Supplementary table 1

Name of oligonucleotides	Sequence
mtDNA forward	CCTATCACCCTTGCCATCAT
mtDNA reverse	GAGGCTGTTGCTTGTGTGAC
nuclear DNA forward	ATGGAAAGCCTGCCATCATG
nuclear DNA reverse	TCCTTGTTGTTCAGCATCAC
