# Supplementary material for: Metabolic requirements of NK cells during the acute response against retroviral infection
Source: Nat Commun. 2021 Sep 10;12:5376. doi: 10.1038/s41467-021-25715-z (PMC8433386; doi:10.1038/s41467-021-25715-z)
Supplement: Supplementary file 3 — Reporting Summary [file 41467_2021_25715_MOESM3_ESM.pdf]

nature research

Corresponding author(s): Elisabeth Littwitz-Salomon, David K. Finlay

Last updated by author(s): 16.07.2021

Reporting Summary

Nature Research wishes to improve the reproducibility of the work that we publish. This form provides structure for consistency and transparency in reporting. For further information on Nature Research policies, see our Editorial Policies and the Editorial Policy Checklist.

Statistics

For all statistical analyses, confirm that the following items are present in the figure legend, table legend, main text, or Methods section.

n/a	Confirmed	
☐	☒	The exact sample size (n) for each experimental group/condition, given as a discrete number and unit of measurement
☐	☒	A statement on whether measurements were taken from distinct samples or whether the same sample was measured repeatedly
☐	☒	The statistical test(s) used AND whether they are one- or two-sided<br>*Only common tests should be described solely by name; describe more complex techniques in the Methods section.*
☒	☐	A description of all covariates tested
☒	☐	A description of any assumptions or corrections, such as tests of normality and adjustment for multiple comparisons
☐	☒	A full description of the statistical parameters including central tendency (e.g. means) or other basic estimates (e.g. regression coefficient) AND variation (e.g. standard deviation) or associated estimates of uncertainty (e.g. confidence intervals)
☐	☒	For null hypothesis testing, the test statistic (e.g. F, t, r) with confidence intervals, effect sizes, degrees of freedom and P value noted<br>*Give P values as exact values whenever suitable.*
☒	☐	For Bayesian analysis, information on the choice of priors and Markov chain Monte Carlo settings
☒	☐	For hierarchical and complex designs, identification of the appropriate level for tests and full reporting of outcomes
☒	☐	Estimates of effect sizes (e.g. Cohen's d, Pearson's r), indicating how they were calculated

Our web collection on statistics for biologists contains articles on many of the points above.

Software and code

Policy information about availability of computer code

Data collection	Flow cytometry data were collected with BD DIVA 8.0.
Data analysis	Data were analyzed with FlowJo Version 10, Prism Version 8, LIMMA version 4.1, Phantasus Web Platform version 1.12.0 and Microsoft Excel 2016

For manuscripts utilizing custom algorithms or software that are central to the research but not yet described in published literature, software must be made available to editors and reviewers. We strongly encourage code deposition in a community repository (e.g. GitHub). See the Nature Research guidelines for submitting code & software for further information.

Data

Policy information about availability of data

All manuscripts must include a data availability statement. This statement should provide the following information, where applicable:

- Accession codes, unique identifiers, or web links for publicly available datasets
- A list of figures that have associated raw data
- A description of any restrictions on data availability

The data sets supporting the findings of this study are available within the paper and publicly available: GEO accession code-GSE39555GSE39555

Field-specific reporting

Please select the one below that is the best fit for your research. If you are not sure, read the appropriate sections before making your selection.

☒ Life sciences ☐ Behavioural & social sciences ☐ Ecological, evolutionary & environmental sciences

For a reference copy of the document with all sections, see nature.com/documents/nr-reporting-summary-flat.pdf

Life sciences study design

All studies must disclose on these points even when the disclosure is negative.

Sample size	In all experiments a minimum of 3 mice we used per group. In our experience, groups of three mice have been shown to be ideal to work with and to give a first trend between groups. Individual mice can be viewed in all bar graphs throughout the manuscript - each dot corresponds to one mouse.
Data exclusions	Data were excluded if there was a contamination with non-NK cells in isolated NK cells fraction (in vitro killing assay, one naive sample was contaminated and excluded)
Replication	Experiments were repeated at least twice (except 6j, 7e-h) with similar results.
Randomization	Naive mice were randomly chosen from boxes with 5 mice. Mice for infection were randomly chosen from boxes with naive mice. After infection, randomization was done for mHep treatment
Blinding	Some researchers participating in experiments were blinded to group allocation

Reporting for specific materials, systems and methods

We require information from authors about some types of materials, experimental systems and methods used in many studies. Here, indicate whether each material, system or method listed is relevant to your study. If you are not sure if a list item applies to your research, read the appropriate section before selecting a response.

Materials & experimental systems

n/a	Involved in the study	
☐	☒	Antibodies
☐	☒	Eukaryotic cell lines
☒	☐	Palaeontology and archaeology
☐	☒	Animals and other organisms
☒	☐	Human research participants
☒	☐	Clinical data
☒	☐	Dual use research of concern

Methods

n/a	Involved in the study	
☒	☐	ChIP-seq
☐	☒	Flow cytometry
☒	☐	MRI-based neuroimaging

Antibodies

Antibodies used	Zombie Aqua (1:1000, 423102, BioLegend), CD3 (17A2, FITC, 1:200, 100204, BioLegend), CD11b (M1/70, PECy7, 1:400, 101216, BioLegend), CD27 (LG.3A10, PE, 1:200, 558754, BD Pharmingen), CD49b (DX5, APC-Vio 770, 1:200, 130-105-249, Miltenyi Biotech), CD69 (H1.2F3, PerCP-Cy5.5, 1:200, 561931, BD Pharmingen), CD71 (R17217, APC, 1:200, 17-0711-82, eBioscience), CD98 (RL388, PE, 1:200, 12-0981-81, eBioscience), cMyc (D84C12, PE, 1:100, 14819, CellSignaling), FasL (MFL3, PerCP-eFluor 710, 1:200, 46-5911-82, eBioscience), GzmB (NGZB, PECy7, 1:200, 25-8898-82, eBioscience), IFNγ (XMG1.2, APC, 1:100, 554413 BD Pharmingen), KI-67 (REA183, PE-Vio770, 1:200, 130-120-419, Miltenyi Biotech), NK1.1 (PK136, BV421, 1:200, 108732, BioLegend), Ter119 (TER-119, BV510, 1:200, 116237, BioLegend), TNFα (MP6-XT22, PE Cy7, 1:100, 25-7321-82, eBioscience), peroxidase-conjugated goat anti-mouse antibody (1:400, 115-035-003, Dianova).
Validation	Antibodies are all well described and validated (https://www.biolegend.com/, https://www.bdbiosciences.com/, https://www.thermofisher.com/, https://www.miltenyibiotec.com/). Antibodies were validated for murine cells via flow cytometry. For new antibodies, titrations were performed. Fluorescence minus one (FMO) controls were performed for experiments.

Eukaryotic cell lines

Policy information about cell lines

Cell line source(s)	FBL-3 cells: Institute for Virology, University Hospital Essen, University of Duisburg-Essen, Germany; YAC-1 cells: School of Immunology and Biochemistry, Trinity College Dublin, The University of Dublin, Dublin, Ireland

Authentication	None of the cell lines used were authenticated from the authors
Mycoplasma contamination	All cell lines were tested negative for mycoplasma contamination
Commonly misidentified lines (See ICLAC register)	No commonly misidentified lines were used in the study

Animals and other organisms

Policy information about studies involving animals; ARRIVE guidelines recommended for reporting animal research

Laboratory animals	C57BL/6 mice, male and female, age between 6-12 weeks
Wild animals	The study did not involve wild animals
Field-collected samples	The study did not involve field-collected samples
Ethics oversight	Animal Research Ethics Committee (AREC) at Trinity College Dublin and Irish Health Products Regulatory Authority (HPRA)

Note that full information on the approval of the study protocol must also be provided in the manuscript.

Flow Cytometry

Plots

Confirm that:

- [x] The axis labels state the marker and fluorochrome used (e.g. CD4-FITC).
- [x] The axis scales are clearly visible. Include numbers along axes only for bottom left plot of group (a 'group' is an analysis of identical markers).
- [x] All plots are contour plots with outliers or pseudocolor plots.
- [x] A numerical value for number of cells or percentage (with statistics) is provided.

Methodology

Sample preparation	Preparation of single cell suspension using cell strainers
Instrument	BD FACS Canto II
Software	BD DIVA 8.0 and FlowJo Version 10
Cell population abundance	After bead isolation of NK cells, purity of isolated NK cell fraction was approximately 85-96%. Purity was determined by flow cytometry.
Gating strategy	Lymphocytes (FSC-A, SSC-A) -> singlets (SSC-H, SSC-W) -> viable cells (Zombie negative) -> non T cells (CD3-) -> NK cells (NK1.1+ CD49b+)

- [x] Tick this box to confirm that a figure exemplifying the gating strategy is provided in the Supplementary Information.
